# Heart Rate Variability Moderates the Association Between Beliefs About Worry and Generalized Anxiety Disorder Symptoms

**DOI:** 10.3389/fnins.2020.569359

**Published:** 2020-10-07

**Authors:** Grace M. Fishback, Lyvia Chriki, Julian F. Thayer, Michael W. Vasey

**Affiliations:** ^1^Department of Neurology, University of Colorado School of Medicine, Denver, CO, United States; ^2^Private Practice, Newton, MA, United States; ^3^Department of Psychological Science, School of Social Ecology, University of California, Irvine, Irvine, CA, United States; ^4^Department of Psychology, The Ohio State University, Columbus, OH, United States

**Keywords:** heart rate variability, worry, generalized anxiety disorder, worry beliefs, cognitive control capacity

## Abstract

Paradoxically, some individuals who experience pathological worry also have good capacity for top-down control over their thoughts. Why such individuals would nevertheless worry excessively remains unclear. One explanation is suggested by research showing that those experiencing pathological worry are set apart from healthy controls by their beliefs that worry has utility and that effective worrying requires them to consider all possibilities before terminating a worry bout. This suggests that worriers with good capacity for cognitive control may engage in prolonged worry because they believe it is adaptive to do so. In a sample of 109 college students, among whom individuals reporting pathological worry were overrepresented, we tested this hypothesis using an objective index of top-down control capacity (i.e., resting vagally mediated heart rate variability [vmHRV]) and self-report measures of beliefs about worry and generalized anxiety disorder (GAD) symptom severity/status. As predicted, GAD symptom severity and vmHRV interacted to predict beliefs about worry. Specifically, high GAD symptoms were most strongly associated with beliefs that worry has utility at *higher* levels of vmHRV. Furthermore, this pattern was mostly a function of the belief that worry serves to distract the worrier from more emotional things. Similarly, high GAD symptoms were most strongly associated with endorsement of an ‘as many as can’ (AMAC) problem-solving rule when vmHRV was *high*. From the opposite perspective, both worry utility beliefs and AMAC rule endorsement were associated with the highest GAD symptom severity at higher levels of vmHRV. This was also true for the belief that worry distracts from more emotional things predicting analog GAD status. These results suggest that worriers who have higher levels of top-down control capacity may initiate and persist in worry, at least initially, because they value it. However, why they nevertheless rate their worry as excessive and uncontrollable is an important question for future research.

## Introduction

Generalized anxiety disorder (GAD), is characterized by uncontrollable and excessive worry (i.e., pathological worry; American Psychiatric Association [APA], 2013). It is common, debilitating, and persistent over many years (Kessler et al., 2012). Furthermore, many individuals with GAD fail to respond to current treatments and those who do respond often fail to maintain improvement over several years (e.g., Cuijpers et al., 2014), suggesting there may be sources of heterogeneity that moderate treatment response. One candidate domain of heterogeneity is the capacity for top-down control over cognition (Toh and Vasey, 2017; Vasey et al., 2017). Understandably, scholars have linked pathological worry to deficits in such cognitive control (e.g., Borkovec et al., 1983; Hirsch and Mathews, 2012). However, studies of top-down control capacity in worriers and individuals with GAD reveal substantial heterogeneity in their results. For example, evidence suggests that such individuals vary widely in their self-reported levels of attentional control (AC) or, more broadly, the dimension of effortful control (EC; see Vasey et al., 2017). Whereas some studies have found significant negative correlations between GAD status/symptoms and self-reported AC/EC and similar constructs (e.g., Armstrong et al., 2011; Olatunji et al., 2011), others have found no association (e.g., Bienvenu et al., 2004) or even a significant positive association (e.g., Rosellini and Brown, 2011).

Studies using behavioral measures of top-down control also present a mixed picture. Although individuals with GAD sometimes perform worse than controls on tests of AC and cognitive flexibility (e.g., Olatunji et al., 2011; Stefanopoulou et al., 2014), other studies have found no difference (e.g., Hoehn-Saric et al., 1989). Indeed, in two separate studies, Yiend et al. (2014) found individuals with GAD to be significantly *faster* than controls in disengaging attention from threat cues. Consistent with such mixed findings, Derryberry and Reed (2002) found that high trait-anxious college students reporting high AC failed to show the difficulty disengaging attention from threat cues seen among their low AC counterparts. Lonigan and Vasey (2009) found similar results in a youth sample.

Neuroimaging studies also reveal heterogeneity in both structural and functional measures of brain regions involved in cognitive control among pathological worriers. For example, Makovac et al. (2016a) and Carnevali et al. (2019) found individuals with GAD to have lower average gray matter volume than healthy controls in regions of the PFC involved in top-down control. However, in contrast, Mohlman et al. (2009) found medial orbital PFC volume to be *positively* associated with scores on the Penn State Worry Questionnaire (PSWQ). Makovac et al. (2016b) found individuals with GAD had lower functional connectivity than healthy controls at baseline between the amygdala and regulatory regions of the PFC. In contrast, Etkin et al. (2009) found individuals with GAD to show atypical *heightened* functional connectivity at rest between the amygdala and the dorsolateral PFC, a region that is associated with cognitive control. Similar heterogeneity is seen in response to tasks involving processing of negative stimuli. For example, Price et al. (2011) found individuals with GAD to show hypoactivity in the PFC compared to controls during an emotional Stroop task. In contrast, Makovac et al. (2016b) found that functional connectivity between the amygdala and regulatory areas in the PFC *increased* among individuals with GAD following a perseverative cognition induction.

Psychophysiological studies measuring vagally mediated heart rate variability (vmHRV) also reveal heterogeneity among pathological worriers. As articulated in the Neurovisceral Integration Model (NIM; Thayer and Lane, 2000) and Polyvagal Theory (Porges, 2008), measures of vmHRV provide an index of activity in the parasympathetic nervous system, which is associated in turn with activity in brain regions and circuits involved in inhibitory control (Lane et al., 2009; Nugent et al., 2011; Thayer et al., 2012). For example, higher vmHRV at rest predicts better performance on tasks requiring top-down control such as the think/no-think task, which requires control over memory retrieval (Gillie et al., 2014), and the thought-suppression paradigm, which requires control over ongoing thought (Gillie et al., 2015). Furthermore, studies show that higher vmHRV is associated specifically with better capacity to regulate attention with respect to threat-stimuli. For example, higher vmHRV predicts greater ability to disengage attention from fearful face distractors (Park et al., 2013) and better inhibition of return to fearful versus neutral faces (Park et al., 2012).

Unsurprisingly given such findings, studies have linked low resting vmHRV to pathological worry (e.g., Thayer et al., 1996; Carnevali et al., 2019). A meta-analysis by Chalmers et al. (2014) shows that individuals with GAD do indeed have lower resting vmHRV on average than controls (Hedge’s g = −0.55). However, even an effect of such magnitude leaves more than 75% overlap between groups. Thus, it is not surprising that some studies have failed to find a difference (e.g., Kollai and Kollai, 1992; Hammel et al., 2011; Aldao and Mennin, 2012; Fisher and Newman, 2013; Levine et al., 2016). Studies comparing high and low worriers have produced similar variability, with some studies finding the expected difference (e.g., Brosschot et al., 2007), others finding no difference (e.g., Knepp and Friedman, 2008; Mankus et al., 2013) and at least one finding high worriers to have significantly *higher* vmHRV at rest than low worriers (Davis et al., 2002 [study 2]). The high end of the range of vmHRV scores in Mankus et al.’s (2013) analog GAD group (absolute value of mean successive differences [|*MSD|*] range = 4.09–170.38) versus their low GAD symptoms group (|*MSD|* range = 4.58–82.41) illustrates the presence of individuals with high vmHRV among those high in GAD symptoms.

Given that some individuals reporting high levels of GAD symptoms also have high capacity for cognitive control, we must ask why such individuals nevertheless experience excessive worry. One explanation is that they intentionally initiate and persist in worry because they believe it serves primary adaptive goals (Freeston et al., 1994). Specifically, such individuals may believe that worry has positive effects [e.g., enhanced problem-solving (Davey, 1994)], or that it fosters avoidance of or preparation for anticipated catastrophic outcomes (Davey et al., 1996; Cartwright-Hatton and Wells, 1997). Consistent with this view, Borkovec and Roemer (1995) interviewed individuals with GAD and identified six beliefs about functions served by worry. Specifically, the GAD group tended to believe that worry can (1) enhance motivation to complete tasks, (2) aid in problem-solving, (3) help one prepare for the worst, (4) aid in planning to avoid negative events, or (5) serve to distract from more anxiety-provoking thoughts. Sixth, they tended to hold the superstitious belief that worrying about something makes it is less likely to happen or at least feel that way.

It is easy to see how worriers might come to regard worry as serving such functions. Since feared outcomes rarely happen, their non-occurrence following a period of worry may negatively reinforce worry as a coping strategy (Davey and Meeten, 2016). Similarly, worry can be reinforced by virtue of its ability to blunt autonomic arousal (Borkovec et al., 2004) or foster avoidance of aversive emotional contrasts (Newman and Llera, 2011). Furthermore, if worriers believe that worry helps them prepare for the worst and they are able to handle feared outcomes better than they expected when they do occur, then worry can seem effective even if they would have weathered the event just as well without worrying. As noted by Freeston et al. (1994), such beliefs may help explain why worriers continue to worry even though it is an aversive experience. One implication of this is that worriers who hold such beliefs and who have good cognitive control ability may channel that capacity toward persisting in worry despite its unpleasantness. Similarly, worriers who have good cognitive control but who believe that worry has utility may feel it would be bad to try to limit their worrying (Cartwright-Hatton and Wells, 1997).

Such beliefs set those experiencing pathological worry apart from controls. A review by Hebert et al. (2014) showed that worry utility beliefs characterize individuals diagnosed with GAD (e.g., Borkovec and Roemer, 1995; Ladouceur et al., 1998; Newman and Llera, 2011), GAD-analogs (e.g., Freeston et al., 1994), and high worriers (e.g., Davey et al., 1996; Laugesen et al., 2003). Furthermore, such beliefs are associated with higher levels of worry in response to stressful events (Iijima and Tanno, 2013). They may also interfere with readiness for change in therapy. In a highly anxious community sample, Covin et al. (2008) found that positive beliefs about worry were significantly negatively associated with readiness for change. Similarly, Laberge et al. (2000) found that worry decreased when positive beliefs about worry were targeted in cognitive-behavior therapy (CBT) for GAD. Importantly, they found that the more positive beliefs changed, the more change in worry severity was seen over time.

Evidence suggests that worriers especially regard worry as a way of regulating anxiety through distraction. In two studies comparing GAD analogs (i.e., individuals who met diagnostic criteria for GAD based on questionnaire responses) to controls, Borkovec and Roemer (1995) found that those high in GAD symptoms were especially characterized by the belief that their worries effectively distract them from even more emotional things. Indeed, only that belief significantly differentiated the analog GAD samples from comparison groups in both studies. These “more emotional things” may be images that activate heightened autonomic arousal symptoms (Borkovec et al., 2004) or they may be unpredictable contrasting spikes in negative emotion (Newman and Llera, 2011). Given its distinctiveness, the current study included a special focus on this belief.

Beyond holding beliefs in worry’s utility, pathological worriers are set apart from controls by their problem-solving orientation (Davey et al., 1992; Freeston et al., 1994). Not only are they unusually likely to rate worry as useful for problem-solving (Ladouceur et al., 1998), they also tend to believe that such a purpose is best served when they consider as many possibilities as they can when worrying (Davey and Meeten, 2016). That is, they follow an ‘as many as can’ (AMAC) rule when worrying rather than stopping when they no longer ‘feel like continuing’ (FLC). Evidence suggests that following an AMAC rule fosters perseveration during worry whereas following an FLC rule is associated with termination of a worry bout (Davey and Meeten, 2016). We suggest further that adherence to an AMAC rule should especially foster perseverative worry among worriers having good capacity for cognitive control, which permits them to persist in worrying despite its unpleasantness.

In this study, we tested these predictions using a measure of resting vmHRV as an objective index of top-down control capacity. Specifically, we predicted that (1) GAD symptom severity should be most strongly, positively correlated with worry utility beliefs among individuals with high levels of resting vmHRV because they are able to use their capacity for cognitive control in the service of worrying. In contrast, GAD symptoms should tend to be unrelated to such beliefs among those with low levels of resting vmHRV because they should worry excessively mainly because they can’t help it. Furthermore, based on the findings of Borkovec and Roemer (1995), we predicted that (2) this pattern should hold especially for the belief that worry distracts from more emotional things. Similarly, we predicted that (3) GAD symptoms should be most strongly positively correlated with endorsement of an AMAC approach to worry among individuals with high vmHRV. From the opposite point of view, we predicted (4) that beliefs in the utility of worry should be most strongly, positively correlated with GAD symptom severity and GAD status among those with high vmHRV and (5) that should be true especially for the belief that worry distracts from more emotional things. So too did we predict (6) that endorsement of an AMAC rule would be most strongly, positively correlated with GAD severity/status among those with higher levels of vmHRV.

## Materials and Methods

### Participants

Participants were recruited from among students taking introductory psychology at The Ohio State University. Potential participants were screened using the Generalized Anxiety Disorder Questionnaire – IV (GAD-Q-IV; Newman et al., 2002) to maximize the number reporting high levels of GAD symptoms. Specifically, all individuals who endorsed at least four of the five dichotomous items on the GAD-Q-IV were sent an email message inviting them to participate. We additionally invited a random subsample of the remainder to ensure that the sample included the full range of GAD symptoms. This resulted in 58 (47.2%) individuals who met the screening criteria at the time they participated in the study and 65 (52.8%) who did not.

The final sample was drawn from 123 participants who completed at least the first laboratory session in a larger, multi-session study. This maximized our sample size despite attrition in later sessions. Additionally, we limited the current sample to those participants having useable heart rate data. Such data were missing for 14 participants due to equipment failure or experimenter error. This resulted in a final sample of 109 participants, in which 65.1% self-identified as female and age ranged from 18-28 years (*M* = 19.3, *SD* = 2.1). They self-identified primarily as Caucasian (71.6%, African American: 7.3%, Asian American: 6.4%, Latino: 3.7%, Multiple Categories: 5.5%, Other: 5.5%). All participants received course credit for their participation.

### Procedure

Upon arrival in the laboratory, after giving informed consent, participants were fitted with the Polar watch and chest belt through which the ECG signal was recorded. Following completion of a brief neutral computer task, participants sat in a quiet room for 5-minutes before their resting ECG was recorded for 5-minutes. They then completed self-report questionnaires in random order, among which were all measures used in the current study.

### Measures

#### Self-Report Questionnaires

*Generalized Anxiety Disorder Questionnaire – IV* (GAD-Q-IV; Newman et al., 2002): The GAD-Q-IV is a self-report questionnaire assessing the diagnostic criteria for GAD based on the Diagnostic and Statistical Manual of Mental Disorders, 4^th^ edition (DSM-IV; American Psychiatric Association [APA], 1994). It is comprised of five yes/no questions assessing frequency and duration of excessive and uncontrollable worry, a checklist of associated symptoms, an open-ended list of worry topics, and two 9-point Likert scale items (ranging from 0 = “none” to 8 = “severe”) regarding level of interference and distress. The GAD-Q-IV can be scored in several ways. Under the approach used by Newman et al. (2002), several items were skipped if a participant’s worry had not persisted for at least 6-months. However, we chose to have all participants answer all questions and to include them in the total score as suggested by Rodebaugh et al. (2008). We otherwise scored the GADQ-IV according to the Newman et al. (2002) formula and used the resulting continuous score as a measure of GAD symptom severity. This continuous score has good psychometric properties (see Rodebaugh et al., 2008) and had good internal consistency in the current sample (Cronbach’s alpha = 0.79). We also used the approach described by Moore et al. (2014) to identify participants who met *DSM-IV* diagnostic criteria for GAD based on their GAD-Q-IV responses. This resulted in an analog GAD subgroup of 26 participants.

*GAD-7 Scale* (Spitzer et al., 2006). The GAD-7 is a 7-item self-report questionnaire assessing GAD symptom severity. The items are based on the *DSM-IV* diagnostic criteria. Answers are ranked on a 4-point Likert scale from 0 (not at all) to 3 (nearly every day). The GAD-7 demonstrates excellent internal consistency (Cronbach’s alpha = 0.92) and good convergent validity. In the current sample, Cronbach’s alpha was 0.90.

*The Overall Anxiety Severity and Impairment Scale* (OASIS; Norman et al., 2006). The OASIS is a 5-item self-report questionnaire assessing the extent to which individuals experience their anxiety as intrusive and impairing. Answers are rated on a 5-point Likert scale ranging from “None” to “Extreme”. Subjects are asked about the frequency of feeling anxious, intensity of the anxiety, and interference of anxiety in their functioning. Norman et al. (2006) reported that the scale has good convergent validity and good test-retest reliability over a one-month period. In the current sample, Cronbach’s alpha was 0.83.

*Reasons to Worry Questionnaire* (RWQ; Borkovec and Roemer, 1995). The RWQ is a 6-item self-report questionnaire assessing reasons why people may worry. Questions ask about six worry functions: motivation to complete tasks, aids in problem solving, preparation for negative events, avoidance of negative events, distraction from emotional topics, and superstitious effects on feared outcomes. Respondents indicate how much each item applies to them using a 5-point Likert scale ranging from “not at all” to “very much”. GAD status correlates with higher scores on each of the six items (Borkovec and Roemer, 1995). In the current sample Cronbach’s alpha was 0.80.

*Why Worry? Questionnaire* (WWQ; Freeston et al., 1994). The WWQ is a 20-item self-report questionnaire regarding a person’s motivations for worrying. Items pertain to ways in which worry prevents negative outcomes or has positive effects. Respondents rate each item on a 5-point Likert scale ranging from “not at all characteristic of me” to “entirely characteristic of me”. We used the total score to represent overall beliefs about the utility of worry. Freeston et al. (1994) demonstrated the WWQ has good agreement with similar measures as well as good internal consistency. Results from Freeston et al. (1994) also show the WWQ to have good ability to distinguish pathological worriers from healthy controls. In the current sample, Cronbach’s alpha for the total score was 0.91.

*Problem Solving* Inventory (PSI; Heppner and Petersen, 1982). The PSI is a 35 item self-report questionnaire measuring participants’ confidence in their ability to solve problems, their tendency to approach or avoid problem solving, and their perception of their degree of control over emotions and behaviors they achieve during problem solving. Items are answered on a 6-point Likert scale (ranging from “Strongly Disagree” to “Strongly Agree”). For the current study we focused only on item #7, which was used as a measure of endorsement of an AMAC rule. Item #7 reads, “When I have a problem, I think of as many possible ways to handle it as I can until I can’t come up with any more ideas.”

#### Vagally Mediated Heart Rate Variability (vmHRV)

Resting vmHRV was estimated using a 5-minute ECG segment recorded using a Polar RS8000 Running Computer Wristwatch and standard Wearlink chest belt. The Polar watch collects data at a 1000 Hz sampling rate and provides a reliable and valid ECG signal (Quintana et al., 2012). We examined the ECG signal and removed artifacts using the KUBIOS HRV analysis package 2.2 (Tarvainen et al., 2009). KUBIOS was also used to compute the root mean square of successive differences (RMSSD) in intervals between heartbeats, which is a time-domain measure of vagally-mediated (parasympathetic) changes in heart rate (Shaffer and Ginsberg, 2017). Higher RMSSD values indicate higher HRV. Values of RMSSD were natural log transformed to better approximate a normal distribution.^1^

### Data Analytic Strategy

Because the RWQ and WWQ both measure worry utility beliefs and were highly correlated (*r* = 0.65, *p* < 0.001; see Table 1), we chose to consolidate them into a composite Worry Utility Beliefs score, which was created by averaging the standardized total scores from each measure. Similarly, because scores on the GAD-Q-IV, GAD7, and OASIS were highly intercorrelated (see Table 1), we created a composite GAD Symptom Severity score by averaging the standardized total scores from the three measures (Cronbach’s alpha = 0.92).

**TABLE 1:** Zero-order correlations and descriptive statistics.

**Variable**	**GAD-Q-IV**	**GAD7**	**OASIS**	**GAD symptom severity**	**vmHRV**	**RWQ**	**WWQ**	**Worry utility score**	**RWQ item #5**	**WWQ item #2**	**Worry distracts score**	**Mean**	**SD**
GAD-Q-IV	–											6.34	3.69
GAD7	**0.85**	–										6.78	5.29
OASIS	**0.79**	**0.82**	–									6.06	3.55
GAD Composite	**0.96**	**0.96**	**0.83**	–								0.00	1.00
vmHRV [Ln(RMSSD)]	−0.02	0.01	0.02	–0.01	–							3.58	0.98
RWQ	**0.36**	**0.38**	**0.26**	**0.36**	0.04	–						14.38	5.08
WWQ	**0.64**	**0.62**	**0.54**	**0.66**	−0.05	**0.65**	–					49.54	16.12
Worry Utility Score	**0.55**	**0.53**	**0.44**	**0.56**	−0.01	**0.91**	**0.91**	–				0.00	1.00
RWQ Item #5	**0.36**	**0.36**	**0.32**	**0.37**	0.10	**0.54**	**0.45**	**0.54**	–			1.96	1.21
WWQ Item #2	**0.33**	**0.36**	**0.34**	**0.36**	0.08	**0.43**	**0.51**	**0.52**	**0.58**	–		2.34	1.22
Worry Distracts Score	**0.38**	**0.41**	**0.37**	**0.41**	0.10	**0.55**	**0.54**	**0.60**	**0.89**	**0.90**	–	0.00	1.00
PSI Item #7	0.08	0.04	0.15	0.06	0.08	−0.06	−0.08	−0.08	**0.22**	−0.02	0.11	2.81	1.36

*All bold correlations significant at *p* < 0.05.*

As noted previously, Borkovec and Roemer (1995) found that their GAD samples were set apart from their comparison groups by their endorsement of one belief, represented by item #5 on the RWQ (“Worrying about most of the things I worry about is a way to distract myself from worrying about even more emotional things, things I don’t want to think about”). Therefore, we focused specifically on that belief. Because item #2 on the WWQ is very similar (“Worrying about less important things distracts me from more emotional subjects that I don’t want to think about”) and because the two items were strongly correlated (*r* = 0.58, *p* < 0.001; see Table 1), we created a composite “Worry Distracts” score by averaging their scores.

All hypotheses were tested via multiple linear regression (MLR) analyses using SPSS Version 25 for Macintosh. For example, to predict the Worry Utility Beliefs score, the GAD Symptom Severity score, vmHRV (i.e., Ln[RMSSD]), and the GAD Symptom Severity x vmHRV interaction were included in the model. Because statistical power to detect interactions is limited in small sample (McClelland and Judd, 1993), we sought to maximize power by limiting the number of predictors in the model to preserve degrees of freedom.^2^ Regression diagnostics were examined for each analysis to identify cases that might be exerting excessive influence on overall model fit or on individual beta weights. Specifically, we used ±1.0 as a cutoff for standardized Dffits and Dfbeta values for each case (Cohen et al., 2002). No high influence cases were identified in any analysis.

All interaction effects with *p* < 0.10 were probed using the PROCESS utility for SPSS (Hayes, 2013; freely available at http://www.afhayes.com). PROCESS estimates simple slopes at specific values of the moderator. We chose to illustrate all interactions by depicting simple slopes for each predictor at high (+ 1 *SD*) and low (−1 *SD*) levels of the moderator. However, PROCESS also implements the Johnson-Neyman technique for deriving regions of significance for the simple slope of the predictor at all observed values of the moderator (see Hayes, 2013, pp. 307–315). For each interaction we report the region of significance in terms of standard deviations from the mean of the moderator, along with the percentile of the distribution corresponding to the region of significance.

## Results

### Preliminary Analyses

All analyses were conducted using 109 participants having complete vmHRV data (88.6% of the original data set). Those participants also had complete data on the other measures with the exception of the PSQ, which was available for 104 participants because it was added after the study began. In the full sample of 123 participants, vmHRV and PSQ data were missing completely at random (Little’s Missing Completely at Random test *p* = 0.263). According to their GAD-Q-IV responses, 23.9% (n = 26) met *DSM-IV* GAD criteria. Based on the GAD-7, 29.3% (n = 32) scored above the clinical cut-off whereas the OASIS identified 36.7% (n = 40) who fell in the clinical range. Table 1 shows descriptive statistics and correlations for all variables. Notably, GAD symptom severity was uncorrelated with resting vmHRV (*r* = −0.01, *p* = 0.948). Unexpectedly, item #7 of the PSI did not correlate significantly with GAD symptom severity. However, it was significantly positively correlated with the “worry distracts from more emotional things” item (#5) on the RWQ (*r* = 0.22, *p* < *0.05*).

### Primary Analyses

#### Predictions 1 and 2: GAD Symptom Severity Interacts With vmHRV to Predict Worry Utility Beliefs

**FIGURE 1:**
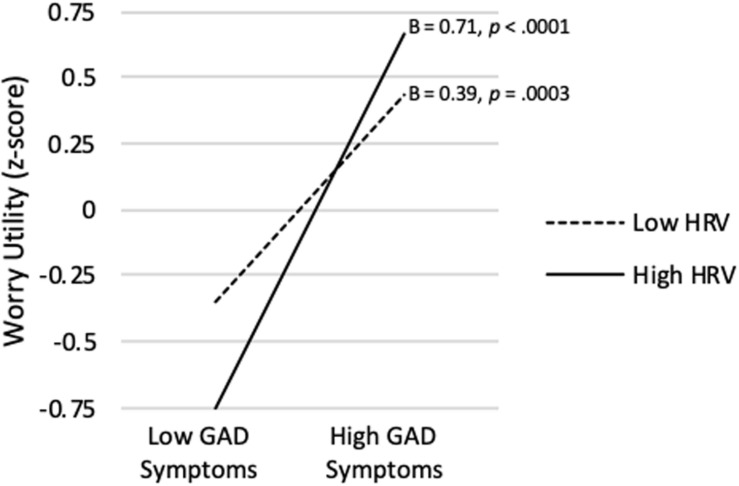
GAD symptom severity predicting worry utility beliefs score at high (+ 1 *SD*) and low (−1 *SD*) vmHRV.

As shown in Table 2, the model predicting Worry Utility Beliefs from GAD Symptom Severity, vmHRV, and their interaction was significant (*R^2^* = *0.327*, p < 0.001). Although GAD Symptom Severity was significantly positively correlated with Worry Utility Beliefs on average (semi-partial r [*sr* = 0.550, *p* < 0.0001), a significant interaction showed that association to be conditional upon level of vmHRV (*sr* = 0.184, *p* = 0.024). As shown in Figure 1, when vmHRV was high (i.e., + 1 *SD*), the simple slope for GAD Symptom Severity was significant (B = 0.71, *p* < 0.0001). When vmHRV was low (i.e., −1 *SD*), the simple slope remained significant but was weaker in magnitude (B = 0.39, *p* = 0.0003). The Johnson-Neyman technique revealed that GAD Symptom Severity was significantly positively correlated with Worry Utility Beliefs except for vmHRV < -1.71 *SD*s (percentile = 8.3). This correlation was strongest when vmHRV was highest.

**TABLE 2:** Summary of regression analyses predicting Worry Utility, Worry Distracts, and ‘As Many As Can.’

	**Dependent variable**							
	Worry utility score (R^2^ = 0.327***)		Worry distracts score (R^2^ = 0.235***)		Worry utility with worry distracts as a covariate (R^2^ = 0.492***)		**‘As Many As Can’ (R^2^ = 0.052)**	
**Variable**	**B (*SE)***	***sr***	**B (*SE)***	***sr***	**B (*SE)***	***sr***	**B (*SE)***	***sr***
Constant	0.000 (0.072)	–	0.000 (0.085)	–	0.001 (0.070)	–	0.008 (0.097)	–
GAD Symptom Composite	0.501*** (0.073)	0.550***	0.424*** (0.085)	0.424***	0.396*** (0.078)	0.357***	0.091 (0.101)	0.088
vmHRV	-0.035 (0.074)	−0.038	0.061 (0.086)	0.061	−0.060 (0.071)	−0.059	0.067 (0.096)	0.068
GAD Composite x vmHRV	0.145* (0.063)	0.184*	0.226** (0.074)	0.234**	0.095 (0.062)	0.107	0.175* (0.084)	0.205*
Worry Distracts Score	–	–	–	–	0.416*** (0.080)	0.364***	–	–

B = unstandardized regression coefficient; *SE* = standard error; *sr* = semi-partial correlation coefficient. ***p < 0.001;*p < 0.01;*p < 0.05; ^†^*p* < 0.10.

**FIGURE 2:**
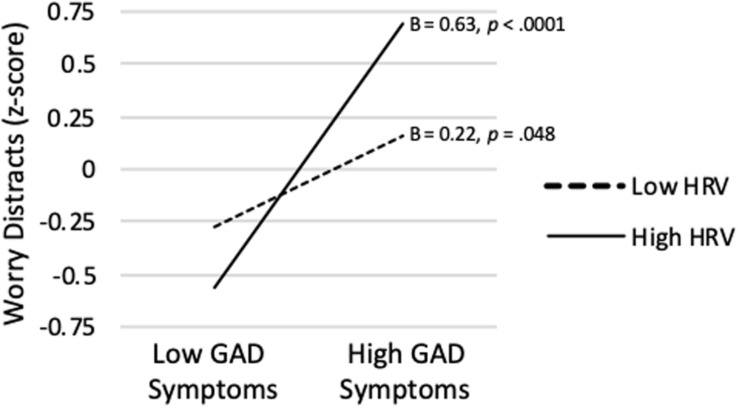
GAD symptom severity predicting worry distracts score at high (+ 1 *SD*) and low (−1 *SD*) vmHRV.

Table 2 also shows that the same model predicting the Worry Distracts score was significant (*R^2^* = *0.235*, p < 0.001). Although GAD Symptom Severity was significantly positively correlated with Worry Distracts on average (*sr* = 0.424, *p* < 0.0001), a significant interaction showed that association varied depending on level of vmHRV (*sr* = 0.234, *p* = 0.007). As shown in Figure 2, when vmHRV was high, the simple slope for GAD Symptom Severity was significant (B = 0.63, *p* < 0.0001). When vmHRV was low, the simple slope remained significant but was weaker in magnitude (B = 0.22, *p* = 0.048). The Johnson-Neyman technique revealed that GAD Symptom Severity was significantly positively correlated with Worry Distracts except for vmHRV < -1.01 *SD*s (percentile = 12.8). This correlation was strongest when vmHRV was highest.

Finally, Table 2 shows that when the Worry Distracts score was entered as a covariate into the model predicting the Worry Utility score, the GAD Symptom Severity x vmHRV interaction became non-significant (*sr* = 0.107, *p* = 0.130). Thus, the variance in the Worry Utility score predicted by the GAD Symptom Severity x vmHRV interaction was accounted for largely by the belief that worry distracts from more emotional things.

### Prediction 3: GAD Symptom Severity Interacts With vmHRV to Predict AMAC Rule Endorsement

**FIGURE 3:**
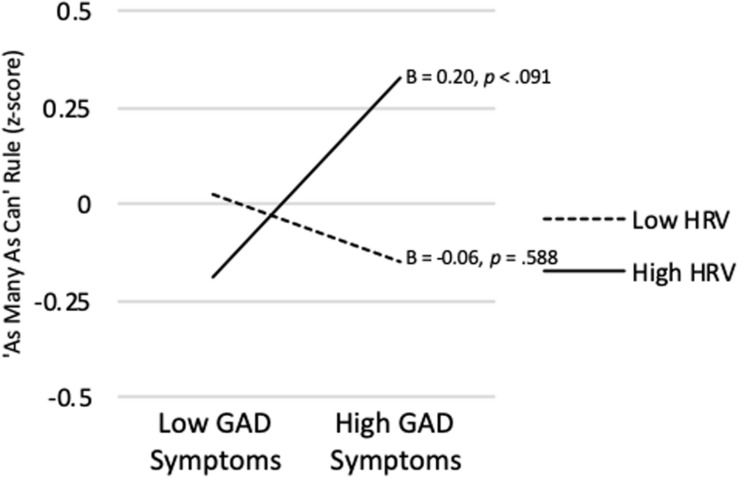
GAD symptom severity predicting ‘As Many As Can’ rule endorsement at high (+ 1 *SD*) and low (−1 *SD*) vmHRV.

Table 2 also shows that the model predicting the AMAC rule from GAD Symptom Severity, vmHRV, and their interaction was not significant (*R^2^* = *0.052*, p = 0.144). Nevertheless, as predicted, the AMAC rule x vmHRV interaction was significant (*sr* = 0.205, *p* = 0.0395). As shown in Figure 3, when vmHRV was high, the simple slope for the AMAC rule was positive and approached significance (B = 0.204, *p* = 0.091). When vmHRV was low, it was non-significant (B = −0.06 *p* = 0.588). The Johnson-Neyman technique revealed that GAD Symptom Severity was significantly positively correlated with strength of AMAC rule endorsement only for vmHRV > 1.15 *SD*s (percentile = 95.2). This correlation was strongest when vmHRV was highest.

#### Predictions 4 and 5: Worry Utility Beliefs Interact With vmHRV to Predict GAD Symptom Severity and Analog GAD Status

**FIGURE 4:**
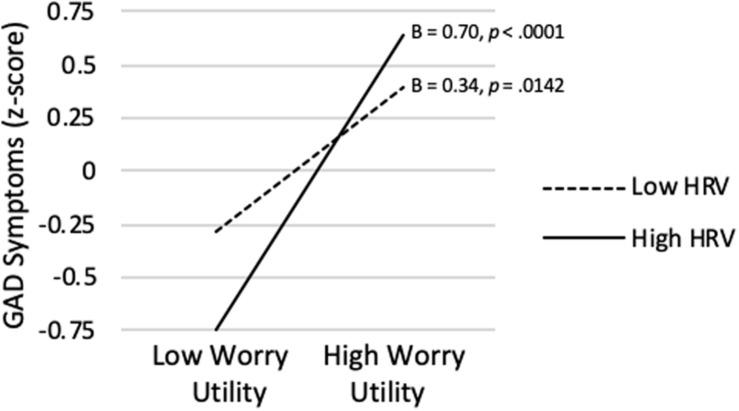
Worry utility beliefs score predicting GAD symptom severity at high (+ 1 *SD*) and low (−1 *SD*) vmHRV.

As shown in Table 3, the model predicting GAD Symptom Severity from Worry Utility Beliefs, vmHRV, and their interaction was significant (*R^2^* = *0.313*, p < 0.0001). Worry Utility Beliefs were significantly positively correlated with GAD Symptom Severity on average (*sr* = 0.516, *p* < 0.0001). Although the Worry Utility Beliefs x vmHRV interaction did not achieve significance (*sr* = 0.142, *p* = 0.083), its pattern was consistent with expectation. As shown in Figure 4, when vmHRV was at + 1 *SD*, the simple slope for Worry Utility Beliefs was significant (B = 0.70, *p* < 0.0001). When vmHRV was at −1 *SD*, the simple slope remained significant but was weaker in magnitude (B = 0.34, *p* = 0.014). The Johnson-Neyman technique revealed that Worry Utility Beliefs were significantly positively correlated with GAD Symptom Severity except for vmHRV < -1.21 *SD*s (percentile = 11.9). This correlation was strongest when vmHRV was highest.

**TABLE 3:** Summary of regression analyses predicting GAD symptom severity.

	**Predictor Variable**					
	Worry Utility (R^2^ = 0.313***)		Worry Distracts (R^2^ = 0.223***)		**‘As Many As Can’ (R^2^ = 0.058)**	
**Variable**	**B (*SE)***	***sr***	**B (*SE)***	***sr***	**B (*SE)***	***sr***
Constant	0.000 (0.081)	–	−0.027 (0.086)	–	−0.031 (0.094)	–
Predictor	0.521*** (0.082)	0.516***	0.370*** (0.088)	0.360***	0.054 (0.095)	0.056
vmHRV	−0.054 (0.082)	−0.050	−0.015 (0.086)	−0.015	0.003 (0.095)	0.003
Predictor x vmHRV	0.176†(0.101)	0.142†	0.268** (0.102)	0.226**	0.218* (0.093)	0.229*

N = 109 except for ‘as many as can’ analysis for which N = 104. B = unstandardized regression coefficient; *SE* = standard error; *sr* = semi-partial correlation coefficient. ***p < 0.001;*p < 0.01;* *p* < 0.05; ^†^*p* < 0.10.

**FIGURE 5:**
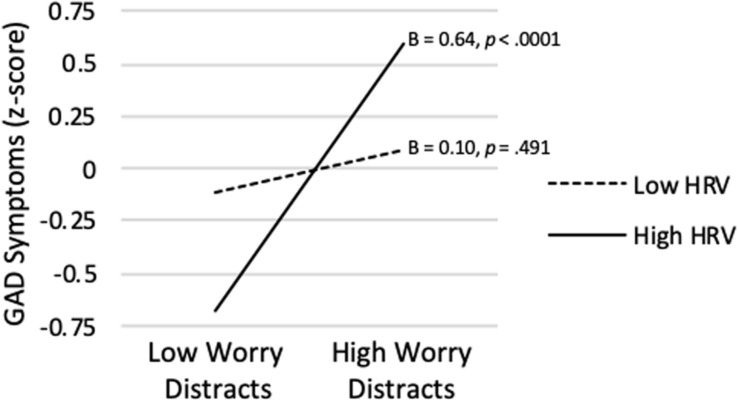
Worry distracts score predicting GAD symptom severity at high (+ 1 *SD*) and low (−1 *SD*) vmHRV.

Table 3 also shows that the model predicting GAD Symptom Severity from Worry Distracts, vmHRV, and their interaction was significant (*R^2^* = *0.223*, p < 0.0001). Although Worry Distracts was significantly positively correlated with GAD Symptom Severity on average (*sr* = 0.360, *p* < 0.0001), a significant interaction term showed that association varied depending on level of vmHRV (*sr* = 0.226, *p* = 0.001). As shown in Figure 5, when vmHRV was high, the simple slope for Worry Distracts was significantly positive (B = 0.64, *p* < 0.0001). The simple slope was non-significant when vmHRV was low (B = 0.10, *p* = 0.491). The Johnson-Neyman analysis revealed that Worry Distracts was significantly correlated with GAD Symptom Severity except for vmHRV < −0.54 *SD’*s (percentile = 18.4). This correlation was strongest when vmHRV was highest.

As shown in Table 4, the binary logistic regression model predicting analog GAD Status from Worry Utility Beliefs, vmHRV, and their interaction was significant (Nagelkerke *R^2^* = *0.447*, p < 0.0001). Worry Utility Beliefs were significantly positively correlated with analog GAD Status on average (*p* = 0.0011). However, the Worry Utility Beliefs x vmHRV interaction did not approach significance (*p* = 0.189) and was not interpreted further.

**TABLE 4:** Summary of binary logistic regression analyses predicting GAD status.

	**Predictor Variable**					
	**Worry Utility (Nagelkerke R^2^ = 0.447, *p* < 0.0001)**		**Worry Distracts (Nagelkerke R^2^ = 0.273, *p* = 0.0001)**		**‘As Many As Can’ (Nagelkerke R^2^ = 0.077, *p* = 0.141)**	
**Variable**	**B (*SE)***	***p-*value**	**B (*SE)***	***p-*value**	**B (*SE)***	***p-*value**
Constant	-1.827 (0.346)	0.0000	-1.490 (0.283)	0.0000	-1.316 (0.251)	0.0000
Predictor	1.760 (0.385)	0.0011	0.889 (0.272)	0.0011	-0.092 (0.253)	0.715
vmHRV	-0.428 (0.329)	0.193	-0.214 (0.240)	0.373	0.078 (0.285)	0.785
Predictor x vmHRV	0.507 (0.386)	0.189	0.676 (0.319)	0.034	0.552 (0.267)	0.039

*N = 109 except for ‘as many as can’ analysis for which *N* = 104. B = unstandardized regression coefficient; *SE* = standard error; coefficients are expressed in a log-odds metric.*

Table 4 also shows that the model predicting analog GAD Status from Worry Distracts, vmHRV, and their interaction was significant (Nagelkerke *R^2^* = *0.223*, p < 0.0001). Although Worry Distracts was significantly positively correlated with analog GAD Status on average (*p* = 0.001), a significant interaction term showed that association varied depending on level of vmHRV (*p* = 0.034). As shown in Figure 7, when vmHRV was high, the simple slope for Worry Distracts was significantly positive (B = 1.57, *p* = 0.0001). The simple slope was non-significant when vmHRV was low (B = 0.21, *p* = 0.620). The Johnson-Neyman analysis revealed that Worry Distracts was significantly positively correlated with analog GAD Status except for vmHRV < -0.42 *SD*s (percentile = 18.3). This correlation was strongest when vmHRV was highest.

### Prediction 6: AMAC Rule Interacts With vmHRV to Predict GAD Symptom Severity and Analog GAD Status

**FIGURE 6:**
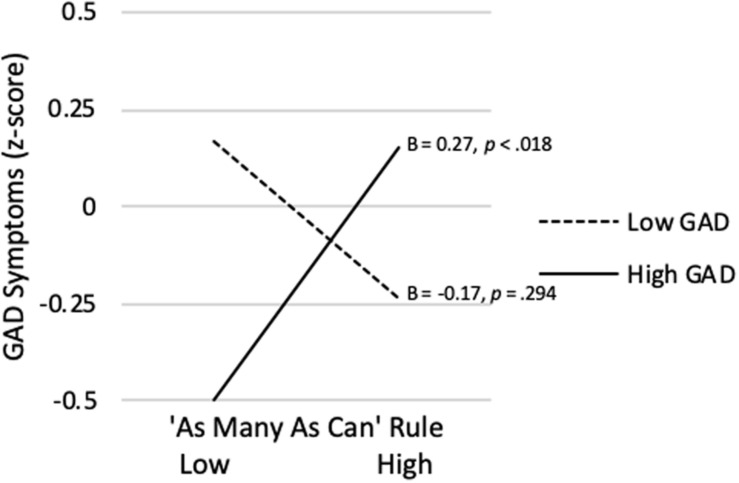
‘As Many As Can’ rule endorsement predicting GAD symptom severity at high (+ 1 *SD*) and low (−1 *SD*) vmHRV.

As shown in Table 3, the model predicting GAD Symptom Severity from the AMAC rule, vmHRV, and their interaction was not significant (*R^2^* = *0.058*, p = 0.112). However, as predicted, the AMAC rule x vmHRV interaction was significant (*sr* = 0.229, *p* = 0.020). The Johnson-Neyman technique revealed that strength of endorsement of the AMAC rule was significantly positively correlated with GAD Symptom Severity only for vmHRV > 0.865 *SD*s (percentile = 88.5). Additionally, the simple slope was significantly negative for vmHRV < -2.76 *SD*s (percentile = 3.85). As shown in Figure 6, when vmHRV at + 1 *SD*, the simple slope for the AMAC rule was significant (B = 0.27, *p* = 0.038). When vmHRV was at −1 *SD*, it was non-significant (B = −0.17, *p* = 0.217).

**FIGURE 7:**
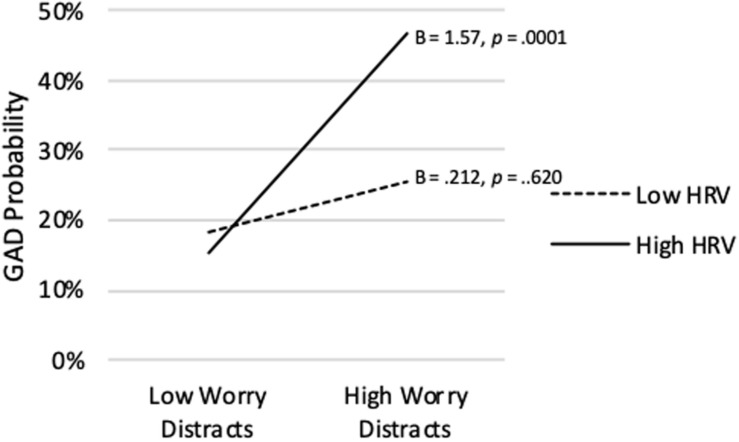
Worry distracts score predicting probability of analog GAD diagnosis at high (+ 1 *SD*) and low (−1 *SD*) vmHRV.

**FIGURE 8:**
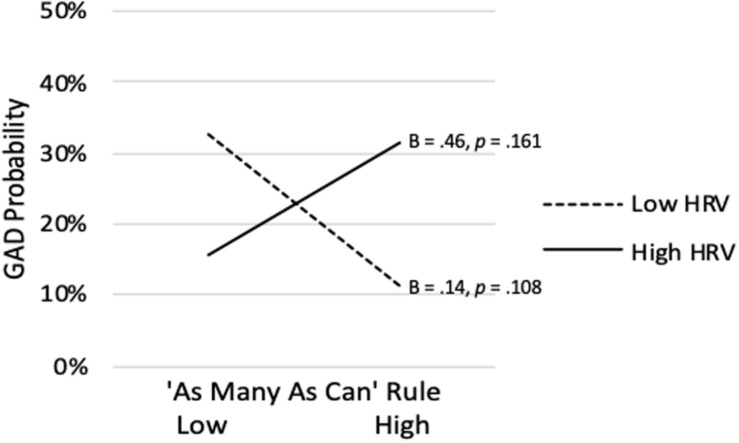
‘As Many As Can’ rule endorsement predicting analog GAD at high (+ 1 *SD*) and low (−1 *SD*) vmHRV.

As shown in Table 4, the model predicting analog GAD Status from the AMAC rule, vmHRV, and their interaction was not significant (Nagelkerke *R^2^* = *0.077*, p = 0.141). However, as predicted, the AMAC rule x vmHRV interaction was significant (*sr* = 0.552, *p* = 0.039). As shown in Figure 8, when vmHRV was at + 1 *SD*, the simple slope for the AMAC rule was positive but not significant (B = 0.455, *p* = 0.161). When vmHRV was at −1 *SD*, it was negative but also non-significant (B = −0.665, *p* = 0.108). The Johnson-Neyman technique revealed that strength of endorsement of the AMAC rule was significantly negatively associated with analog GAD status when vmHRV was low (i.e., <-2.930 SDs; percentile = 2.88). Although this simple slope shifted to a positive association at higher vmHRV, it did not achieve significance.

## Discussion

This study’s aim was to help explain why some individuals experience pathological worry despite having good capacity for top-down control over cognition. We hypothesized that such individuals use that capacity to initiate and persist in worrying despite its aversiveness because they believe it has important benefits and/or they believe in the importance of exhaustively considering all possibilities when worrying. In contrast, such beliefs should be less relevant among worriers having poor cognitive control capacity, who instead should experience excessive worry mainly because they can’t help it.

Our results are consistent with this perspective. GAD symptom severity was indeed most strongly associated with beliefs in worry’s utility when vmHRV was highest. This correlation was weaker at lower vmHRV, becoming non-significant when vmHRV was in the bottom 8.3% of the sample. This pattern was especially apparent for the belief that worry distracts from more emotional things, which research suggests is especially characteristic of pathological worriers (Borkovec and Roemer, 1995). This pattern also emerged when predicting strength of endorsement of an AMAC problem-solving rule. Although the overall regression model in that case was not significant, the interaction term was. Again, the association was strongest when vmHRV was highest. Thus, as expected, worriers are most likely to believe in worry’s utility and endorse an exhaustive approach to problem solving when their resting vmHRV suggests they have good capacity for top-down control of cognition.

In our view these findings suggest that worry utility beliefs and endorsement of an AMAC problem-solving rule foster persistence in worrying among worriers having high capacity for top-down control. That is, we believe that such individuals use their cognitive control capacity to initiate and persist in worrying despite its aversiveness because they believe doing so has important payoffs. Our results support this perspective, especially for the belief that worry is useful because it distracts from more emotional things. Specifically, strong endorsement of that belief predicted the highest GAD symptom severity when vmHRV was *highest*, becoming non-significant when vmHRV was below the 18.4th percentile. Similarly, among those endorsing the strongest belief that worry distracts from more emotional things, the probability of meeting GAD diagnostic criteria based on GAD-Q-IV responses was maximized when vmHRV was *highest*, becoming non-significant when vmHRV was below the 18.3rd percentile. This supports the view of Borkovec and Roemer (1995), that the belief that worry distracts from more emotional things plays a pivotal role in pathological worry. However, our findings suggest further that this is true only for worriers who possess sufficient capacity for top-down control to persist in worrying in an effort to achieve that goal.

This same pattern was also observed for broader worry utility beliefs. Although the hypothesized worry utility belief x vmHRV interaction only approached significance, as expected, such beliefs predicted the highest GAD symptom severity when vmHRV was highest and became non-significant when vmHRV was below the 11.9th percentile. Results were similar but weaker when predicting analog GAD status.

This pattern was also found for the AMAC approach to problem solving. Endorsement of that approach did interact significantly with vmHRV to predict GAD symptom severity. High endorsement of an AMAC approach was significantly positively associated with GAD symptoms only when vmHRV was above the 75th percentile. This association was significantly negative when vmHRV was lower than -2.8 SDs. However, that region of significance applied to only 3.8% of the sample and should be interpreted cautiously. A similar but weaker pattern was found predicting GAD status. Thus, our findings are consistent with the view that an AMAC approach to problem-solving fosters perseverative worry among individuals with high capacity for cognitive control. This is consistent with Meeten et al.’s (2016) finding that AMAC rule endorsement is associated with increased connectivity between the amygdala and PFC during resting state fMRI. Meeten and colleagues interpret that increased connectivity as reflecting attempts by high worriers to engage in goal-directed worry. In this regard it is important to note that higher connectivity between the amygdala and the PFC is associated with higher vmHRV (Sakaki et al., 2016). Higher vmHRV is also linked to higher inhibition of return to threat, which may foster the type of exhaustive search for novel solutions implied by the AMAC rule (Park et al., 2012).

Given our results, we must ask why someone having good cognitive control capacity might nevertheless learn to worry and come to hold such beliefs about its functions and form. One avenue is through parental influences. Specifically, parents who are worriers may encourage their children to worry, reinforce its occurrence, and model its use as a coping strategy (Aktar et al., 2017). In such ways they may inculcate their children with their beliefs about worry’s utility and there tendency to follow an AMAC rule when worrying.

No matter how worriers having good capacity for cognitive control come to worry initially, their beliefs in worry’s utility and in the need to worry exhaustively may be especially likely to strengthen as a result of worrying. Because they are able to persist in worrying despite its aversiveness, they may be more likely to experience reinforcement for worrying, which should, in turn, reinforce their beliefs about its functions and form. For example, the catastrophic outcomes anticipated by worriers rarely occur and when they do, worriers typically weather them better than they feared they might. That may be especially true for worriers having good capacity for top-down control. Therefore, such a worrier may be more likely to be negatively reinforced by virtue of concluding that worrying helped prevent, or prepare them for, a feared event even if the outcome would have been the same had they not worried (Davey and Meeten, 2016). Similarly, worry can be reinforced by virtue of its ability to blunt autonomic arousal (Borkovec et al., 2004) or foster avoidance of aversive emotional contrasts (Newman and Llera, 2011). These outcomes may be more likely if a worrier is able to draw upon their cognitive control capacity to persist in worrying despite its aversiveness (e.g., see Vasey et al., 2017). Such circumstances also seem likely to create the conditions for beliefs about worry’s functions and form to be strengthened through the process of effort justification (Kitayama and Tompson, 2015). By virtue of their belief in worry’s benefits and the importance of worrying exhaustively, such individuals are motivated to initiate and persist in worrying despite its aversiveness. However, that aversiveness should produce strong cognitive dissonance, which can be reduced by increasing one’s commitment to the beliefs in question. Thus, the more such individuals worry, the more they should come to value it and the more firmly they should be committed to the reasons they have learned to worry and the exhaustive manner in which they think worry should proceed.

Our results suggest several questions for future research. First, the defensive stance toward the world that characterize worriers has been linked to lower levels of vmHRV within the NIM (Thayer and Lane, 2000) and Polyvagal Theory (Porges, 2008). Thus, it remains unclear how a worrier can adopt such a stance toward the world and nevertheless exhibit higher levels of vmHRV. A second important question for future research is why worriers with good capacity for top-down control nevertheless report that their worry is excessive and uncontrollable. One possibility is that such worriers may believe it would be bad to try to limit their worrying as suggested by Cartwright-Hatton and Wells (1997), even though they have the capacity to do so. However, whereas that might lead them to worry excessively it would not explain why they perceive worry to be uncontrollable. Instead, high cognitive control worriers may find their worry spinning out of control and proceeding involuntarily because worry depletes the very cognitive control resources they had initially used to persist in worrying. Evidence suggests that worry does indeed deplete such resources (e.g., Hayes et al., 2008; Stefanopoulou et al., 2014). This is also consistent with findings by Levine et al. (2016) showing that whereas individuals with GAD did *not* differ in vmHRV from healthy controls at baseline, they showed greater reductions in vmHRV during worry. Meeten et al. (2016) provide further evidence supporting such a process. Specifically, they found that higher AMAC rule endorsement predicted stronger declines in vmHRV in individuals with GAD following a perseverative cognition induction. Furthermore, research shows that worriers tend to shift from an AMAC rule at the outset of a catastrophizing worry task to an FLC rule at the end of such a task (Davey et al., 2007). However, it is likely that cognitive control resources are required to implement a goal of stopping worrying following such a rule shift. Consequently, since worrying consumes such resources, worriers having sufficient capacity to initially persist in worrying in accordance with the AMAC rule or their beliefs about worry’s utility should find it difficult to stop the process once they no longer feel like continuing. However, it should be noted that Makovac et al. (2016b) found that higher vmHRV at baseline among individuals with GAD predicted weaker declines in vmHRV following a perseverative cognition induction. This suggests that worriers having high cognitive control may be initially protected from worry-induced declines in cognitive control. If so, they may be able to engage in longer bouts of worry before losing control.

### Limitations

Our study had several limitations. First, given the study’s design, we cannot draw firm conclusions regarding the direction of the associations observed or their causal status. Future research should attempt to resolve questions of directionality. Second, generalizability of our results may be limited by the fact that participants were college students characterized by a narrow age range and limited ethnic diversity. Furthermore, although individuals reporting high GAD symptom severity were well-represented in the current sample, future studies should include clinically diagnosed, treatment seeking cases. Third, we lacked information concerning medications that participants were taking that could alter their resting levels of vmHRV. Since some participants reported high GAD symptoms, it is possible that some may have been taking medications for anxiety and/or depression. Thus, it is possible that medication effects may have contributed to our findings. However, insofar as some such medications can reduce vmHRV whereas others can cause it to increase (see Kemp et al., 2014 and Kemp et al., 2016) such effects seem unlikely to account for our findings.

Finally, given our small sample size, statistical power was limited, especially for detecting interactions (McClelland and Judd, 1993). Consequently, some regression models and interaction effects did not achieve significance despite accounting for substantial percentages of variance. This is especially relevant in the case of the AMAC rule, which was assessed using a single questionnaire item. That undoubtedly increased measurement error and thus further reduced power. Since the item asked about consideration of all possible problem solutions rather than specifically about an AMAC approach to worrying, it may also have failed to measure that construct adequately. Past research has shown that AMAC rule endorsement is associated with worry severity (Davey and Meeten, 2016). In this study item #7 on the PSI did not correlate significantly with GAD symptom severity at the zero-order level. However, that correlation was significant at high levels of vmHRV. AMAC rule endorsement is also correlated with worry utility beliefs (Davey and Meeten, 2016). Thus, it is notable that Item #7 on the PSI was significantly correlated with the worry distracts item on the RWQ. However, it is also notable that the item in question did not correlate significantly with our broader measures of worry utility beliefs. Thus, it remains unclear how well item #7 of the PSI represented AMAC rule endorsement. Future research should utilize a more psychometrically sound measure of this construct, such as the Worry Stop Rule Checklist (Davey et al., 2005).

### Conclusion

Our results suggest that worriers who have good top-down control capacity initiate and persist in worry because they value it. However, why they nevertheless rate their worry as excessive and uncontrollable is an important question for future research.

## Data Availability Statement

The raw data supporting the conclusions of this article will be made available by the authors, without undue reservation.

## Ethics Statement

The studies involving human participants were reviewed and approved by Behavioral and Social Sciences Institutional Review Board of The Ohio State University. The patients/participants provided their written informed consent to participate in this study.

## Author Contributions

GF and MV conducted the data analyses and wrote the first draft of the manuscript. LC collected the data and designed the larger study from which the data were drawn. JT contributed to the critical design of the study and assisted with data analysis. All authors provided important intellectual content in revising the manuscript and approved the final version before submission.

## Conflict of Interest

The authors declare that the research was conducted in the absence of any commercial or financial relationships that could be construed as a potential conflict of interest.

## Footnotes

1 We also used the coefficient of variation for RMSSD to take into account differences in heart rate (see de Geus et al., 2019) but results were unchanged. Therefore, we report only the results based on RMSSD.

2 Models. including sex and age as covariates did not substantively alter the pattern of results.
